# Use of a multileaf collimator to increase the field width achievable with a dynamic wedge

**DOI:** 10.1120/jacmp.v7i3.2186

**Published:** 2006-08-24

**Authors:** Ivan A. Brezovich, Richard A. Popple, Jun Duan

**Affiliations:** ^1^ Department of Radiation Oncology The University of Alabama at Birmingham Birmingham Alabama 35294 U.S.A.

**Keywords:** wedge, multileaf collimation, MLC

## Abstract

A method is proposed for generating dynamic wedges spanning the entire field width, defined as the collimator opening in the wedged direction, without changes to existing hardware. The technique approximates the fluence pattern of a dynamic wedge by sequentially closing the leaves of a 120‐leaf multileaf collimator (MLC). Closure times for the individual leaves were derived by extending the segmented treatment table of the dynamic wedge provided by the manufacturer of the linear accelerator. Using film dosimetry, beam properties of MLC wedges were compared to those of conventional dynamic and mechanical wedges. Profiles and isodose lines of the MLC wedge were almost identical to those of the dynamic wedge, and differed only modestly from the mechanical counterparts. Dose inhomogeneity due to the individually closing leaves was not significant. The high‐dose region at the junction between opposing MLC leaves, unavoidable when the field length (i.e., the opening of the collimator in the nonwedged direction) exceeds the maximum leaf extension of 15 cm, was feathered by moving leaf pairs after their closure for the remainder of the irradiation time. Combining the MLC wedge with a regular dynamic wedge to reduce the line of high dose under the leaf junction is under consideration.

PACS number: 87.53.Mr

## I. INTRODUCTION

The dynamic wedge is a technique by which a mechanical wedge is replaced by a moving collimator jaw to produce a similar fluence pattern.^(^ ^1^ ^,^ ^2^ ^)^ The technique eliminates the time‐consuming handling of the heavy metal wedges, thereby increasing patient safety, throughput, and comfort. In addition, dynamic wedges do not change the beam properties (no beam hardening) and do not cause scatter. Hence the dose distribution in a wedged field can be computed by the treatment‐planning system from open beam data, eliminating tedious wedge data taking during commissioning of the accelerator.

To produce a fluence pattern that simulates a given wedge, the jaw motion must follow a specific position‐versus‐dose trajectory, commonly referred to as a “segmented treatment table” (STT). The “extended dynamic wedge” (EDW) of our accelerator (Clinac 21EX, Varian Medical Systems, Milpitas, CA) uses a single segmented treatment table, the “golden segmented treatment table” (GSTT), from which the STT for any wedge angle between 10° and 60° can be derived.

Due to constraints on over‐travel by the X‐ray jaws, the Varian EDW is limited to a symmetric field width of 20 cm. In the asymmetric mode, the moving jaw can travel the full width on its own side plus 10 cm over‐travel past the central ray, to provide a total field width of 30 cm in the wedged direction. However, in some situations, such as treatment of extremity soft‐tissue sarcomas, a wedged field larger than 30 cm is needed. To provide such wide fields, we developed a dynamic wedge based on a multileaf collimator (MLC). MLC‐based wedges have been proposed by other investigators, primarily for the purpose of obtaining any desired wedge orientation with respect to the collimator.^(^ ^3^ ^,^ ^4^ ^)^ The current investigation is aimed at using MLCs to increase the field width (defined as the field dimension in the direction of the wedge) obtainable with our accelerator. Beam profiles, isodose curves, and wedge factors of MLC wedges are presented and compared to their counterparts from mechanical and standard dynamic wedges.

## II. METHODS AND MATERIALS

### A. Rationale

The investigations were done using our unmodified medical accelerator, which was equipped with a 120‐leaf MLC that covered the entire field, measuring $40\times 40\;{\text{cm}}^{2}$ at the isocenter. The two banks of leaves of the MLC were mounted on separate carriages, one for each side of the field. The carriages could move from the fully open position to 2 cm beyond the central ray. The leaves had a range of 15 cm, that is, they could protrude from the carriage by that distance.

In analogy to conventional dynamic wedges, we could have generated a wide wedged field by first moving one of the two carriages over its full range, and then extending the leaves to their maximum. However, this would have limited the field width to 37 cm, and required major modifications of the MLC controller, since current software does not allow carriage motion while radiation is being delivered.

To achieve a dynamic wedge extending over the entire field width with the unmodified accelerator, we sequentially closed the leaves of the MLC, approximating the fluence pattern of a conventional dynamic wedge oriented perpendicular to the direction of leaf motion. We based the closure times of the individual leaves on the GSTT of the dynamic wedge in order to achieve a similar dose distribution. Since the GSTT of the dynamic wedge was available only for the travel range of the independent jaw from Y=−10 cm to Y=+20 cm, we had to expand it to cover the entire field. This was done using the method of Papatheodorou et al.^(5)^ by approximating the GSTT by an analytic expression of the form

(1) $$\text{GSTT}(Y)=P_{1}\;\text{exp}(-(P_{2}Y)/(1+P_{3}Y)),$$

where GSTT(Y) is the relative number of monitor units (MUs) to be delivered when the moving jaw passes the position Y. The parameters $P_{1},P_{2},\;\text{and}\;P_{3}$ were found by fitting the expression through the available points of the GSTT. The fit values were $P_{1}=1.1451,\;P_{2}=0.09\;531\;{\text{cm}}^{-1}$, and $P_{3}=-0.00\;273\;{\text{cm}}^{-1}$ for the 6‐MV beam, and $P_{1}=1.1163,\;P_{2}=0.07\;636\;{\text{cm}}^{-1}$, and $P_{3}=-0.00\;346\;{\text{cm}}^{-1}$ for the 15‐MV beam. We then substituted these fit values into Eq. (1) to define the dose‐versus‐leaf closure trajectory for a 60° MLC wedge for leaf positions ranging from −20 cm to +20 cm. We will refer to this MLC equivalent of the GSTT as “MLC golden segmented treatment table” (MLCGSTT).

The derived segmented treatment table for an MLC wedge having an arbitrary angle θ, ${\text{MLCDSTT}}_{\theta }$, was obtained by adding radiation from an open field to the MLCGSTT of the 60° wedge, that is,

(2) $${\text{MLCDSTT}}_{\theta }(Y)=W_{0^{{}^{\circ}}}\times \text{MLCGSTT}(0)+W_{60^{{}^{\circ}}}\times \text{MLCGSTT}(Y).$$

The weights of the open field, $W_{0^{{}^{\circ}}}$, and that of the wedged field, $W_{60^{{}^{\circ}}}$, were computed using the ratio of tangents method^(1)^:

(3) $$W_{0^{{}^{\circ}}}=(\text{tan}\;60^{{}^{\circ}}-\text{tan}\;\theta )/\text{tan}\;60^{{}^{\circ}}$$

and

(4) $$W_{60^{{}^{\circ}}}=\text{tan}\;\theta /\text{tan}\;60^{{}^{\circ}}.$$

After multiplying by a scaling factor to specify the radiation dose in absolute units, the MLCGSTT was transferred to the accelerator like any other dynamic MLC leaf pattern, except that the radiation beam was turned off while the leaves were in motion.

The dose steps caused by the closing of individual leaves can be estimated from the slope of the MU‐versus‐position curve given by Eq. (2). The slope is steepest for large, negative values of Y, that is, near the toe of the wedge. A numerical evaluation of a 30° MLC wedge, for example, shows a relative MU difference of 4.4% over the 1‐cm leaf width, between points at −17 cm and −18 cm from the central ray. However, because of the gradual radiation falloff due to the penumbra at leaf edges, the steps in the dose should be smaller than the difference in MUs. In the central region of the field the dose steps are further reduced by the 0.5‐cm leaf width.

The effective wedge factor of the MLC‐wedge, MLCEWF, defined as the ratio of the dose at a reference point of the wedged field to the dose of an open field treated with the same number of monitor units, was computed from the segmented treatment table using the method proposed by Papatheodorou et al.^(5)^ The method assumes that the dose at any given point $Y_{P}$ in the field is modified only by the truncation of the beam by the moving jaw. With this assumption,

(5) $$\text{EWF}(Y_{p})=\text{DSTT}(Y_{p})/\text{DSTT}(Y_{\text{stop}}),$$

where $Y_{\text{stop}}$ is the jaw position at the toe of the wedge. Taking leaf transmission into account, the equivalent wedge factor for the MLC wedge at any desired point $Y_{p}$, $\text{MLCEWF}(Y_{P})$, becomes

(6) $$\begin{array}{c} \text{MLCEWF}(Y_{p})=\text{MLCDSTT}(Y_{p})/\text{MLCDSTT}(Y_{\text{stop}})+\\ T\times (1-\text{MLCDSTT}(Y_{p})/\text{MLCDSTT}(Y_{\text{stop}})), \end{array}$$

where $Y_{\text{stop}}$ is the position of the collimator leaf at the toe, and T is the fraction of radiation transmitted through the MLC leaves.

A potential problem with the MLC wedge is the high‐dose region below the leaf junction. For fields shorter than the 15‐cm leaf extension, the high‐dose region can be eliminated by placing the junction beneath the primary collimator jaws. For fields longer than the maximum leaf span, where a junction within the field is unavoidable, one could follow the closing leaves with the Y collimator jaw and thereby eliminate the high‐dose region within the limits of jaw travel. However, since present software does not allow simultaneous jaw and MLC motion, we created a leaf sequence in which the junction was feathered by moving it linearly across the field during radiation delivery. The distance over which the junction may be feathered is determined by the difference between the extension limits of opposing leaf pairs and the field length. For example, for a 15‐cm leaf extension the junction of a 20‐cm‐long field may be feathered over 10 cm. For a 25‐cm‐long field the feathering distance is reduced to 5 cm, whereas a 30‐cm‐long field cannot be feathered at all.

### B. Experimental verification

Wedge profiles and isodose distributions were measured using film (Kodak Ready Pack V) in a polystyrene phantom. For wedge profile measurements, the film was placed perpendicular to the beam axis at a depth of 5 cm. Because of the limited film size $\left(40.9\times 32.8\;{\text{cm}}^{2}\right)$, the film had to be placed at 95 cm focus‐to‐film distance, with a source‐to‐surface distance (SSD) of 90 cm. For isodose curves, the film was placed into the beam axis and was exposed edge‐on, with the phantom surface at 90 cm SSD. Measurements were performed for MLC wedges, EDWs, and the mechanical wedges. Wedge factors were measured with the aid of a 0.125 cm^3^ ionization chamber (Model TM31002‐0755, PTW, Freiburg, Germany) and an integrating electrometer. The ionization chamber was placed at the isocenter, at the depth of dose maximum $\left(d_{\max}\right)$ in a water phantom. Treatment times were read from the treatment computer screen.

## III. RESULTS

**Figure 1:**
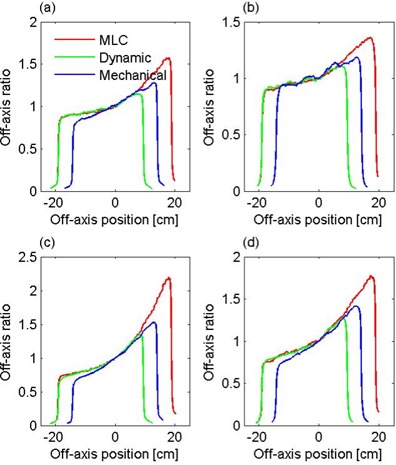
Beam profiles of 15° wedges at (a) 6 MV and (b) 15 MV, and of 30° wedges at (c) 6 MV and (d) 15 MV. The field length is 14 cm for all wedges, whereas the field width is the maximum that can be achieved with each wedge.

Comparisons of beam profiles produced by the MLC, the EDW, and the corresponding mechanical wedges are shown in Fig. 1. Nominal wedge angles of 15° and 30° were investigated, at beam energies of 6 MV and 15 MV. In each case, the dimension in the unwedged direction was 14 cm, whereas the field width was the largest available with the given wedge. The MLC profiles agree well with those of the EDW, but the profiles of the hard wedges show some discrepancy. Dose inhomogeneities due to the 1‐cm‐wide leaves are clearly discernible, especially near the toe of the 30° wedge at 6 MV. However, due to the blurring effect by the penumbra at the leaf edges, the dose along the wedge profile does not deviate by more than about ±1% from a smooth line.

**Figure 2:**
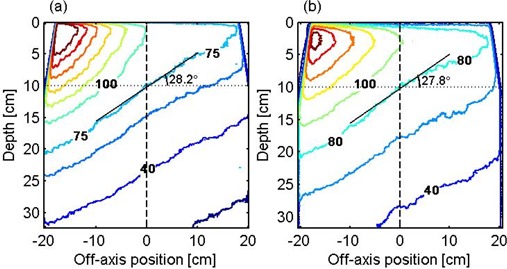
Isodose lines of a $40\times 14\;{\text{cm}}^{2}$ field produced by a 30° MLC wedge at (a) 6 MV and (b) 15 MV. Isodose lines are 40%, 60%, the isodose line passing through 10 cm depth at the central axis, 100%, 120%, 140%, 160%, 180%, and 200%.

An example of isodose lines at 6 MV is shown in Fig. 2 for an MLC wedge with a nominal angle of 30°. Wedge angles were determined, using the definition given in ICRU 24,^(6)^ as the angle between the isodose line passing through the central beam axis at a depth of 10 cm and a line perpendicular to the central axis. To minimize the effect of film noise, a least‐squares fit of a straight line extending 5 cm on either side of the central ray was drawn through points on the isodose line. The wedge angles, determined in this manner, are summarized in Table 1.

**Table 1:** Wedge angles produced by the multileaf collimator wedge

Energy (MV)	Nominal wedge angle (°)	Measured wedge angle (°)
6	15	17
15	15	14
6	30	29
15	30	28

**Figure 3:**
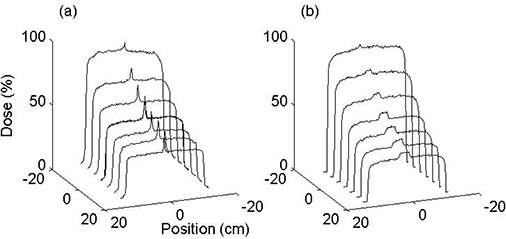
Beam profiles of a $40\times 25\;{\text{cm}}^{2}$ field produced by a 15° MLC wedge at 6 MV at various distances from the beam axis. The profiles were measured along the nonwedged direction in a plane perpendicular to the beam axis at 5 cm depth. (a) When the leaves remain stationary after closure, a narrow region of substantial dose enhancement is generated. (b) Moving the leaf junction during irradiation results in a relatively wide area of small dose enhancement.

Figure 3 shows beam profiles of a $40\times 25\;{\text{cm}}^{2}$ field taken at $d_{\max}$ across the wedged direction. Leakage through the rounded leaf ends and the small gap between them manifests itself as peaks near the center of the profiles. The effect is most pronounced at the heel end of the wedge, with an estimated 45% enhancement in dose. The peaks diminish toward the toe due to the shorter time during which the leaves remain closed. Feathering greatly reduces the height of the peaks but increases their width.

Wedge factors of MLC wedges were computed with and without consideration of leaf penetration. Leaf penetration was taken as 1.7% at 6 MV and 1.9% at 15 MV. These values were measured at 7 cm at 6 MV and 10 cm at 15 MV using a $20\times 14\;{\text{cm}}^{2}$ field and a large ion chamber that extended over several leaves, and so are averages that include intraleaf and interleaf leakage. The computed wedge factors computed at $d_{\max}$ for a $40\times 14\;{\text{cm}}^{2}$ field are compared to measurements taken under the same conditions (Table 2). Agreement between measurements and calculations is very close when the leaf transmission is taken into account.

**Table 2:** Computed and measured central axis wedge factors

Nominal wedge angle (°)	Energy (MV)	Computed neglecting leaf transmission	Computed including leaf transmission	Measured
10	6	0.680	0.692	0.685
15	6	0.578	0.588	0.589
15	15	0.686	0.699	0.692
30	6	0.390	0.407	0.400
30	15	0.502	0.512	0.511

To determine the effect of the MLC wedge on treatment time, we programmed the accelerator to deliver doses of 100 MU, 200 MU, and 300 MU at various field sizes and 6 MV and 15 MV beam energy. For comparison, we delivered the same number of MUs using the EDW and a mechanical wedge. Table 3 summarizes the results for the 30‐cm field width, the largest one achievable with all wedges, and the 10‐cm field length. Treatment times at other field sizes for the MLC wedge and the dynamic wedge (where available) are shown in Table 4. Since treatment times for mechanical wedges are independent of field size, the results are the same as in Table 3. Based on the time it takes to close individual leaves, the increase in treatment time compared to a conventional wedge can be estimated knowing that the maximum leaf speed is 2.5 cm/s. For a field length of 10 cm, each leaf has to travel a distance of 11 cm to close (1 cm for overlap), thus requiring 4.4 s. Since 59 leaves have to close when a full‐field MLC wedge is delivered, the treatment time should be extended by an extra 4.33 min.

**Table 3:** Times (min) to deliver 100 MU, 200 MU, and 300 MU with various wedges at a nominal dose rate of 400 MU/min

Nominal wedge angle	Energy	MLC wedge $30\times 10\;{\text{cm}}^{2}$ MU			Dynamic wedge $30\times 10\;{\text{cm}}^{2}$ MU			Mechanical wedge $30\times 10\;{\text{cm}}^{2}$ MU		
(°)	(MV)	100	200	300	100	200	300	100	200	300
10	6	1.82	2.33	2.80	0.69	0.89	1.09	0.25	0.5	0.75
10	15	1.43	2.19	3.17	0.70	0.90	1.11	0.25	0.50	0.75
15	6	1.86	2.51	2.98	0.67	0.84	1.03	0.25	0.50	0.75
15	15	1.72	2.51	3.01	0.69	0.87	1.06	0.25	0.50	0.75
30	6	2.11	2.77	3.01	0.62	0.76	0.94	0.25	0.50	0.75
30	15	2.16	2.76	2.99	0.63	0.77	0.94	0.25	0.50	0.75

**Table 4:** Times to deliver 100 MU with a 15° MLC wedge and a similar dynamic wedge at 6 MV and a nominal dose rate of 400 MU/min to fields of various sizes

		Delivery time (min)	
Field width (cm)	Field length (cm)	MLC wedge	Dynamic wedge
8	4	1.19	0.35
14	8	1.12	0.43
10	10	1.02	0.37
20	5	0.99	0.51
20	10	1.66	0.52
30	5	1.14	0.67
30	10	1.94	0.67
40	5	1.24	N/A
40	10	2.21	N/A

The smaller than expected treatment times shown in Table 3 are due to the simultaneous motion of groups of up to four neighboring leaves in the low‐dose region at 100 MU. The leaf motion calculator was apparently programmed to move several leaves when dose steps amounted to ≤1 MU. When larger doses were given, the extra time due to the MLC wedge was longer because fewer leaves were closing simultaneously.

## IV. DISCUSSION AND CONCLUSION

Wedged fields of 40 cm width at the isocenter can be generated by sequentially closing the leaves of an MLC. The steps in the dose due to the closing of individual leaves are insignificant. At field lengths of 14 cm or less, dose distributions produced by the MLC wedges are very similar to those of standard dynamic wedges. The extra treatment time required by the closing of leaves should not preclude the clinical use of MLC wedges, especially if the alternative is a multifield treatment that includes patient shifts. The MLC can be used for field lengths of up to about 25 cm if some dose enhancement below the junction of opposing leaves is acceptable.

Leakage through individual leaves and between neighboring leaves increases the dose compared to that delivered by a jaw‐based dynamic wedge, which has negligible penetration. It increases the wedge factor (see Table 2) and decreases the wedge angle by adding dose preferentially near the heel of the wedge. However, this extra dose does not lead to inaccurate treatments since the treatment‐planning system takes leakage into account so that proper adjustments of beam weights and other parameters can be made. Alternatively, one could use a segmented treatment table that compensates for leaf penetration by slightly earlier closure of individual leaves, especially those near the heel of the wedge.

Dose spikes at the junction of opposing leaves in fields longer than 14 cm could be substantially reduced by relatively simple changes in accelerator software. One could program an independent X‐ray jaw to follow the closing MLC leaves within the limits of jaw travel, or combine a dynamic wedge with an MLC wedge. The dose spikes in the remaining 10 cm of field width should be relatively shallow, since the leaves near the toe remain open for most of the irradiation time. Alternatively, one could increase the field length up to the full 40‐cm collimator opening by using two or three carriage groups, similar to large‐field intensity‐modulated radiotherapy treatments. Dose spikes would not be problematic if one selected field lengths of less than 14 cm for each carriage group, so that all leaf junctions would be covered by X‐ray jaws. The increase in delivery time caused by the closing of individual leaves could be nearly eliminated if the dose were delivered using the sliding window technique in which many leaves move simultaneously. MLC wedges could also be extended beyond the 30° angles that were the subject of our investigation, although we feel that 40‐cm‐wide fields at larger wedge angles would not be clinically useful.

To clinically implement the MLC wedge, we envision storage of a precalculated MLCGSTT for a 60° wedge in the MLC controller, similar to the GSTT of conventional dynamic wedges. Smaller wedge angles would be generated by adding an open field with the appropriate weight. Patient‐specific MLC‐shaped fields could be designed using an appropriate treatment‐planning system. Similar to the rectangular fields that we investigated, the MLC controller would interrupt the beam and close leaves according to the MLCGSTT. Quality assurance tests would be similar to those for conventional dynamic wedges. One would need to measure only a small number of beam profiles to ensure that the precalculated MLC control files are not corrupted.

As suggested by Leavitt et al.^(3)^ and Zhu,^(4)^ a sliding window technique could be used to generate wedges at any desired rotation angle with respect to X‐ray jaws. This would greatly reduce the need to turn the collimator, and may even allow the design of accelerators having fixed collimators. Eliminating collimator rotation would greatly simplify the design of accelerators, increase precision, and reduce the number of quality assurance tests for the medical physicist. Although the limited range of current MLC leaves restricts the usefulness of the concept, it could be quite practical in conjunction with longer leaf travel within a carriage position.
